# Co-Translational Protein Folding and Sorting in Chloroplasts

**DOI:** 10.3390/plants9020214

**Published:** 2020-02-07

**Authors:** Fabian Ries, Claudia Herkt, Felix Willmund

**Affiliations:** Molecular Genetics of Eukaryotes, University of Kaiserslautern, Paul-Ehrlich-Strasse 23, 67663 Kaiserslautern, Germany; riesf@rhrk.uni-kl.de (F.R.); herkt@rhrk.uni-kl.de (C.H.)

**Keywords:** chloroplast gene expression, protein synthesis, molecular chaperones, protein targeting, translocation

## Abstract

Cells depend on the continuous renewal of their proteome composition during the cell cycle and in order to replace aberrant proteins or to react to changing environmental conditions. In higher eukaryotes, protein synthesis is achieved by up to five million ribosomes per cell. With the fast kinetics of translation, the large number of newly made proteins generates a substantial burden for protein homeostasis and requires a highly orchestrated cascade of factors promoting folding, sorting and final maturation. Several of the involved factors directly bind to translating ribosomes for the early processing of emerging nascent polypeptides and the translocation of ribosome nascent chain complexes to target membranes. In plant cells, protein synthesis also occurs in chloroplasts serving the expression of a relatively small set of 60–100 protein-coding genes. However, most of these proteins, together with nucleus-derived subunits, form central complexes majorly involved in the essential processes of photosynthetic light reaction, carbon fixation, metabolism and gene expression. Biogenesis of these heterogenic complexes adds an additional level of complexity for protein biogenesis. In this review, we summarize the current knowledge about co-translationally binding factors in chloroplasts and discuss their role in protein folding and ribosome translocation to thylakoid membranes.

## 1. Introduction

During protein synthesis, the linear genetic information is decoded into proteins, the versatile macromolecules that contribute to nearly all biological pathways. In this process, unfolded nascent polypeptides emerge into an extremely dense subcellular environment, which can reach protein concentrations of 300–400 g/L [1]. Due to their unfolded nature and the exposure of hydrophobic amino acid segments, nascent polypeptides are highly prone for aggregation and the formation of unwanted interactions with other proteins. Thus, cells require an efficient protein biogenesis network that provides a high degree of coordination and safeguarding. The tasks of nascent polypeptide biogenesis encompass enzymatic processing of their N-termini, the prevention of premature folding, the translocation of precursors across membranes and the final folding and assembly into tertiary and quaternary conformations, reviewed in [1,2,3,4,5]. It is now clear that protein folding starts already inside the ribosomal exit tunnel, which provides sufficient space for the formation of small alpha-helices, beta-hairpins or zinc-finger domains [6,7,8]. At the ribosomal tunnel exit site, a number of biogenesis factors directly associate with ribosomes in order to bind emerging nascent chains, which assist in folding, translocation, maturation and early quality control, reviewed in [3,4,9]. This event of nascent polypeptide binding and release is highly orchestrated and follows a well-ordered cascade. First steps of nascent chain maturation involve enzymatic modification such as removal of the formyl residue at the N-terminal methionine by peptide deformylases in bacteria and organelles, or excision of the initiator methionine and N-terminal acetylation in eukaryotes [10,11]. Co-translational translocation of nascent polypeptides requires targeting of translating ribosomes to subcellular membranes such as the endoplasmic reticulum, reviewed in [12], the mitochondrial outer membrane [13] and the thylakoid membranes of chloroplast (see below) in eukaryotic cells or the inner membrane of bacterial cells for protein secretion and membrane integration. Again, this translocation is mediated via ribosome-associated proteins. Co-translational folding is performed by molecular chaperones that are structurally remarkably different between prokaryotic and eukaryotic cells. Binding and release of molecular chaperones during de novo folding follows a highly orchestrated cascade shaping the energy landscape along the folding trajectory of the nascent polypeptide reviewed in [2,3,4,9].

In plant cells, protein synthesis occurs in the three subcellular compartments cytosol, chloroplasts and mitochondria, which demands for a high degree of cross-talk and co-ordination [14,15,16,17]. Chloroplasts contain a small semi-autonomous genome which encodes for less than a hundred proteins. Importantly, most of these chloroplast-encoded proteins are important subunits of the essential macromolecular chloroplast complexes performing protein synthesis or maintaining gene expression [18]. Consistent with the prokaryotic origin of chloroplasts, gene expression shows many hallmarks of bacterial gene expression. However, plastids also acquired several unique features. These aspects have been covered recently by several excellent reviews, e.g., [19,20,21,22]. Importantly, many molecular chaperones and factors involved in nascent polypeptide processing and sorting are also found in chloroplasts [10,23,24,25]. However, the understanding about the molecular mechanisms and general principles is by far less understood compared to the advanced knowledge which accumulated for bacterial cells or the cytosolic systems. In the following, we review what is currently understood about co-translationally acting factors that serve folding and sorting within plastids. We briefly summarize the current knowledge about the orthologous system in bacteria before we highlight the current data available describing chloroplast processes. For the parallel biogenesis of imported proteins and further downstream processes, such as biogenesis of thylakoid membranes and the assembly of the major complexes involved in photosynthesis, we refer to excellent reviews in the field, e.g., [26,27,28,29,30,31].

## 2. The Ribosome Associated Molecular Chaperone Trigger Factor

**Figure 1:**
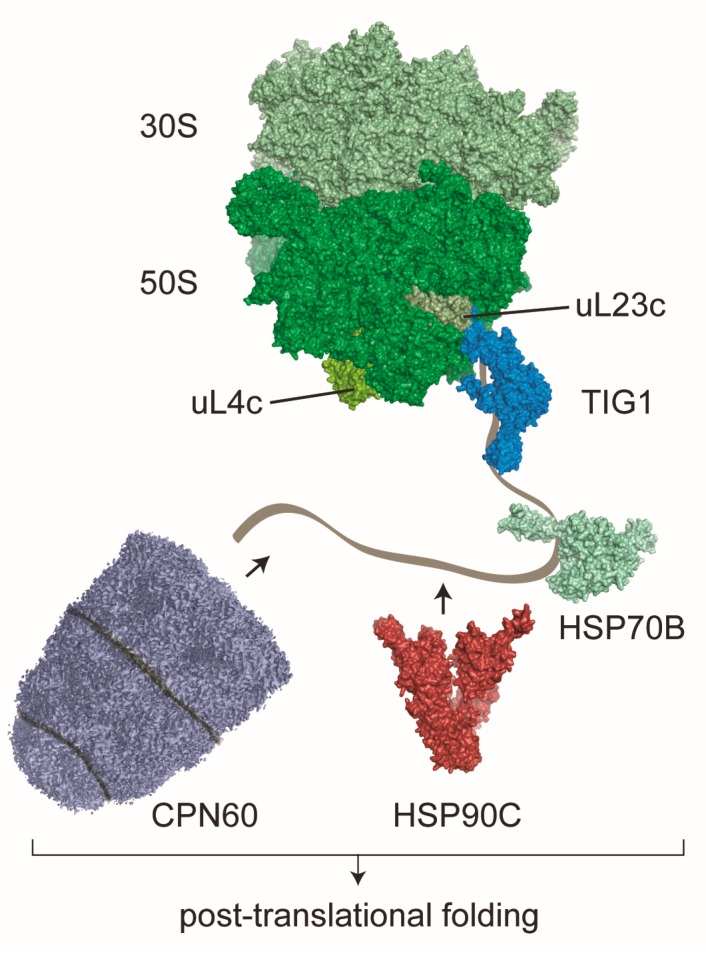
The putative network of molecular chaperones serving co-translational folding in chloroplasts. Comparable to bacteria, chloroplasts contain the dragon-shaped chaperone trigger factor (TIG1), which co-translationally associates with translating 70S ribosomes. Trigger factor binds near the ribosomal exit tunnel at uL23c via a ribosome binding motif. This motif is strongly conserved between bacteria and higher plants and shows less conservation in algae. Additional chaperones that were found to bind translating ribosomes in chloroplasts are the DnaK homolog HSP70B with co-chaperones, the dimeric HSP90C and the chaperonin CPN60. CPN60 consists of a tetradecamer forming two stacked rings and a heptameric lid of the CPN20 family, which encapsulates substrates in the folding chamber of CPN60. HSP70B, HSP90C and CPN60 are also majorly involved in downstream post-translational folding and the maturation of imported chloroplast-localized proteins. Structural models are based on [52] (ribosome), [43] (TIG1), PDB 4B9Q and [53] (HSP70B), PDB 2O1U (HSP90C) and [54] (CPN60).

In prokaryotes, trigger factor (TF) is the most prominent molecular chaperone that directly contacts the ribosome for receiving emerging nascent polypeptides (Table 1). TF was first discovered in 1987 as a protein that “triggers” the folding of nascent polypeptides. In this study, TF associated with chemically denatured outer membrane protein (OMP) OmpA of *Escherichia coli* (*E. coli*) to promote an assembly-competent folding of OmpA [32]. Later, the same group found that TF binds to ribosomes in a 1:1 ratio and that TF is one of the most abundant proteins in *E. coli* cells, even exceeding the abundance of ribosomes [33]. In the last two decades, TF was intensively studied and arguably became the best understood molecular chaperone reviewed in [3,4,34]. TF consists of three domains in a dragon-shaped conformation that directly binds at the 50S ribosomal polypeptide tunnel exit site (Figure 1), which perfectly situates the molecular chaperone for its task of binding nascent polypeptides [35,36]. In *E. coli*, TF binds the majority of emerging nascent polypeptides, including most cytosolic proteins, periplasmic proteins and OMPs [37]. This also explains the cellular requirement of 2–3 fold molar excess TF over ribosomes [33] in order to provide sufficient chaperones for global protein synthesis of cells. Interestingly, ribosome profiling studies showed that TF is not pre-bound to ribosomes but that it rather binds once the first 60–70 amino acids of a newly synthesized protein have emerged from the exit tunnel [37]. Thereby, modifying enzymes have sufficient time and space for performing the task of processing the N-terminus of nascent polypeptides. Unlike many other molecular chaperones, substrate binding and release of TF is ATP-independent. Substrate binding involves several putative non-polar binding sites along the full length of an extended cavity within the TF molecule that interact with hydrophobic segments of the unfolded substrates [38,39]. Although deletion of *E. coli* TF shows no obvious growth defect under ambient temperatures [40,41], the chaperone function seems to be important for promoting de novo folding of newly-synthesized proteins. Through the co-translational engagement of TF, nascent polypeptides are prevented from premature folding and the chaperone even unfolds local domain structures that formed early during protein synthesis. In fact, TF seems to protect partially folded states within a nascent chain by preventing unwanted distal interactions of this section and thereby reshaping the energy landscape during folding which makes overall folding more efficient [42].

In eukaryotic cells, genes encoding trigger factor can only be found in organisms that have plastids, i.e., plants and algae, suggesting an exclusive role of trigger factor in chloroplasts but not in mitochondria (Table 1). However, compared to the advanced knowledge about bacterial TF, we are just beginning to understand its role in plastids. In the genomes of algae, only a single gene encoding trigger factor (TIG1) can be found. In contrast, mosses and land plants contain at least two *TIG* genes that are thought to derive from a gene duplication early in land plant evolution [43,44]. *TIG1* encodes a trigger factor protein harboring all bona fide domains (the N-terminal ribosome binding domain, the peptidyl-prolyl cis-trans isomerase middle domain and the C-terminal chaperone module [34]) whereas TIG2 seems to be a truncated version most likely containing only an extended ribosome binding domain [43]. The sequence conservation between chloroplast TIG1 and TF of *E. coli* is rather low (~18% identity) and even shares only 24% identity between TIG1s of algae and land plants (i.e., *Chlamydomonas reinhardtii* and *Arabidopsis thaliana*, respectively) [43]. However, structural comparison through low resolution small-angle X-ray scattering showed that the overall conformation of TIG1 molecules is strikingly conserved between green algae, land plants and bacteria [43]. Expression and protein levels in *A. thaliana* leaves suggest that TIG1 accumulates at higher amounts compared with TIG2 [44,45]. Unlike TF of *E. coli*, chloroplast TIG1 is not present in molar excess to ribosomes but rather comprises just ~10% of the molar concentration of chloroplast ribosomes. This, together with the finding that only 2%–5% of chloroplast-localized TIG1 molecules bind to ribosomes, indicates that the chaperone is not required for folding of all chloroplast-encoded proteins [46]. Direct ribosome binding of TIG1 and TIG2 still awaits proof but there is evidence that the interaction might be similar in chloroplasts and bacteria (Figure 1). Interestingly, TIG1 seemed to have evolved a more specific function in chloroplasts, since TIG1 of *C. reinhardtii* and *A. thaliana* are both not able to substitute their counterpart in bacteria, unlike other plastidic chaperones such as co-chaperones of HSP70B or CPN60 which are able to complement the respective bacterial mutants [46,47,48,49]. This might be the consequence of a lower ribosome-binding affinity or their narrower substrate specificity compared with the broad affinity of bacterial trigger factor [46]. However, chloroplast TIG1s share a certain substrate binding specificity with *E. coli* TF, also binding to peptides with short hydrophobic segments [46]. Deletion or reduction of chloroplast TIG1 results in subtle phenotypes with growth defects occurring upon prolonged dark exposure of *C. reinhardtii tig1* mutants and reduced linear electron flow rates of photosynthesis in *C. reinhardtii* and *A. thaliana tig1* mutants. This points toward an altered energy budgeting in the absence of the chaperone [46]. One explanation for this effect is, that chloroplast translation seems upregulated in *C. reinhardtii tig1* mutants. This could be an energy costly reaction to compensate misfolding and degradation of newly synthesized proteins upon lack of the chaperone in plastids [46,50]. All these findings point to an important role of TIG1 during chloroplast biogenesis. It remains to be shown, which chloroplast nascent polypeptide requires TIG1 for folding and how TIG2 in higher plants or other chaperones may compensate a loss of TIG1. Furthermore, chloroplast TIG1 might exhibit additional functions outside the ribosomal context similar to putative roles of bacterial TF in complex assembly [51]. However, these non-translational functions are not fully understood to date.

## 3. Other Co-Translationally Acting Chaperones in Chloroplasts

Upon the first binding of bacterial TF at the ribosome, newly synthesized polypeptides are triaged to two additional major chaperone systems, the Hsp70 DnaK and the chaperonin machinery GroEL/ES, reviewed in [2,3,4]. Each of these chaperone systems exhibit a distinct function within the de novo folding cascade but both systems are able to at least partially take over the others’ chaperones function, which provides an important safety network for protein homeostasis. For example, overexpression of GroEL with its co-chaperonin GroES allows cells to compensate the loss of TF and DnaK [55]. DnaK recognizes and binds short exposed hydrophobic peptides within its substrates. DnaK may act co- or post-translationally, together with its co-chaperone DnaJ and the nucleotide exchange factor GrpE. At least 15% of all newly synthesized proteins are thought to be DnaK substrates during de novo folding in *E. coli* [40,41,56]. Here, DnaK promotes native folding by preventing aggregation and by disentangling unwanted associations of unfolded polypeptides.

The large GroEL machinery is constituted of two stacked rings, consisting of seven GroEL, which encapsulate protein substrates within a dedicated folding chamber. This folding chamber is sealed by the ATP-dependent binding of the heptameric co-chaperonin GroES. GroEL/S folds at least 10% of all newly-synthesized proteins of *E. coli* cells and is believed to generally act post-translationally, reviewed in [2,57]. However, there is evidence in literature that GroEL may even act co-translationally since the complex was found in association with bacterial polysomes. This interaction was abolished when nascent polypeptides were released from translating ribosomes by addition of puromycin [58]. The authors speculated that GroEL may bind emerging nascent polypeptides via its apical domain, and that the substrate is fully encapsulated for folding once it is released from the ribosome. Both, the DnaK/J/E and the GroEL/ES systems play important roles during de novo folding and the refolding of damaged proteins during stress responses, reviewed in [2]. In contrast, chaperones of the eukaryotic cytosol seem to be organized in two distinct functional networks that are either dedicated to stress response or the folding of newly synthesized proteins, respectively [59]. The latter network, termed CLIPS (chaperones linked to protein synthesis) includes HSP70s and its co-chaperones, the nascent polypeptide-associated complex NAC, the Prefoldin complex and the chaperonin TRiC/CCT (Table 1) [59].

Consistent with its prokaryotic origin, chloroplasts are equipped with a chaperone family that is homolog to their bacterial counterpart [23]. In a recent proteomic study of isolated chloroplast ribosomes from *C. reinhardtii* cells, the chaperonin CPN60 with its co-chaperonins, HSP70B with its co-chaperone CDJ1 and HSP90C were described to bind co-translationally. CPN60, HSP70B and HSP90C were also shown to comigrate with chloroplast ribosomes in sucrose gradients in a puromycin-dependent fashion, pointing to direct binding of emerging nascent polypeptides (Figure 1, Table 1) [60]. Thus, even for the maturation of the less than hundred chloroplast-encoded proteins, plastids seemed to have evolved a broad co-translational folding network which might be even more advanced compared with the situation in *E. coli*. Future studies need to elaborate in more detail how the folding task is shared between the different chaperones. For CPN60, one of the most prominent and best-studied substrates is the highly abundant large subunit of the Ribulose-1,5-bisphosphate carboxylase/oxygenase (RbcL), reviewed in [61,62]. It can thus be speculated that CPN60 might receive nascent RbcL already during translation in order to facilitate targeted maturation and RubisCO complex assembly.

## 4. Co-Translational Targeting of Chloroplast-Encoded Proteins to Thylakoid Membranes

In prokaryotic and eukaryotic cells, the early recruitment of protein targeting factors and their competition with ribosome-associated chaperones is essential for efficiently targeting proteins to their respective subcellular destination. Nobel prize-winning Günther Blobel and colleagues showed almost 40 years ago that the signal recognition particle (SRP) is required for targeting membrane or secretory proteins to the endoplasmic reticulum (ER) in eukaryotes and for protein transport to the plasma membrane in prokaryotes [63,64,65,66]. The pathways and contributing components differ between organisms, but their overall function or mechanism is conserved in all domains of life. The mammalian SRP is a ribonucleoprotein consisting of six polypeptides (SRP9, 14, 19, 54, 68, 72—numbers denoting their molecular weight) and a 7S RNA, whereas in *E. coli* only Ffh (homologue of SRP54) and a 4.5S RNA are found (Table 1), reviewed in [67,68]. Protein synthesis and translocation or insertion across or into membranes is strictly coupled in both mammalian and prokaryotic cells to avoid misfolding of hydrophobic proteins within the cytosol or periplasm. Therefore, eukaryotic SRP54 in complex with the SRP 7S RNA binds close to the ribosomal protein L23 located near the peptide exit channel [69] and recognizes N-terminally-located signal sequences on nascent chains emerging from the ribosome. Signal sequences share common structural features like a positively charged N-terminal domain, a hydrophobic core and a polar C-terminal region with a cleavage site [70,71,72,73,74]. Eukaryotic SRP contains SRP9 and SRP14, forming a heterodimer, which is bound to 7S RNA and arrests elongation [75]. It was long thought that such an arrest does not take place in prokaryotes, but it was shown in cryo-EM studies that a translational slowdown occurs at least in *B. subtilis* [76]. It is possible that only larger SRP structures combined with RNAs are able to perform this as SRP needs to stretch over a vast part of the ribosomal surface, a task that possibly only eukaryotic, archaeal and some bacterial SRPs can perform [77,78,79]. In yeast, translational slowdown occurs due to non-optimal codon usage and not because of SRP interaction [80], a mechanism that is still not fully resolved to date. For many years it was stated that SRP binds to signal peptides emerging from the ribosome, but some studies reported that in *E. coli* and yeast SRPs recognize their targets even before the signal sequence leaves the peptide exit tunnel [81,82,83]. These data remain debated and other studies showed that SRP binding still seems to be dependent on signal sequence exposure [84,85]. Nevertheless, SRP bound to ribosome-nascent chain complexes (RNCs) guides them to membranes where an interaction with its receptor (FtsY in *E. coli*, SRα and membrane-anchored SRβ in mammalian cells) takes place. The co-translational import or insertion of a protein can occur via a translocon or in concert with an insertase in the case of internal membrane proteins. Both SRP54/Ffh and its receptor are GTPases. In *E. coli*, FtsY is not an integral membrane protein but loosely associated with it [86,87,88]. The two components (SRP54/Ffh and their receptors) activate each other in a reciprocal, GTP-dependent way [89,90,91], which leads to GTP hydrolysis and a release of SRP. Translation continues at the membrane while the nascent polypeptide passes through the translocon or is guided by an integrase. In *E. coli*, the translocon consists of SecYEG with the integrase YidC, reviewed in [25,92]. The complete SRP targeting is highly conserved amongst organisms but at the same time there are many major differences.

**Figure 2:**
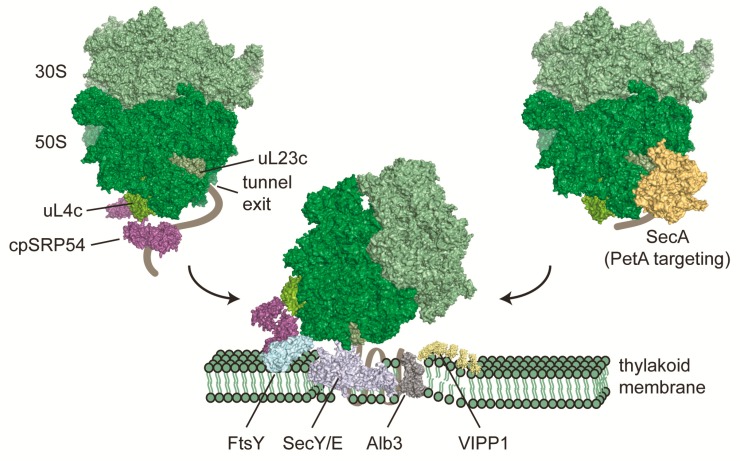
Co-translational targeting of chloroplast ribosome nascent chain complexes to thylakoid membranes. More than 30% of all proteins which are encoded by the chloroplast genome are integral components of thylakoid membranes. Insertion of several of these proteins occurs co-translationally via the cpSRP54 pathway and leads to recruitment of translating ribosomes to thylakoid membranes once the first transmembrane segment emerges from the ribosomal tunnel. Chloroplast SRP54 seems to bind ribosomes via the M domain to the uL4c ribosomal protein. Once this complex is translocated to the membrane, cpSRP54 docks to the translocon by direct interaction with FtsY, adjacent to the SecY/E pore. Membrane integration is assisted by Alb3. VIPP1 may assist integration by providing local areas of protein biogenesis [121]. A parallel co-translational translocation pathway, at least for the integration of Cytochrome *f*, is mediated via SecA and the SecY/E translocon. Structural models are based on [52] (ribosome), PDB 3DM5 and 5L3R (cpSRP54), PDB 5L3R (FtsY), PDB 3J45 and 4V6M (SecY/E), PDB 6AL2 (Alb3), and 4YS0 (SecA).

Chloroplasts contain just another yet specifically adjusted translocation machinery, which shares some features with the bacterial system. A major task of the plastidic translocon system lies in the biogenesis of the ~350 integral proteins of the thylakoid membrane. These proteins are components of the key complexes involved in the central photosynthetic energy-generating process of plant cells. An additional challenge is the integration of chloroplast-encoded proteins together with subunit proteins that are of cytosolic origin and post-translationally imported across the chloroplast envelope, reviewed in [25,93,94,95,96]. Chloroplasts possess a homologue of SRP54, called cpSRP54 but the RNA which is usually associated with the SRP machinery is absent in higher plants (Table 1) [97,98,99,100]. Another surprising feature is the additional, and plastid specific, protein cpSRP43, which associates with cpSRP54 during the post-translational integration of nuclear-encoded light-harvesting chlorophyll a/b binding proteins (LHCPs) into the thylakoid membranes in higher plants [101,102]. Such a post-translational targeting pathway of SRP54 is unique and only found in chloroplasts so far. Interestingly, plastid-specific cpSRP43 does not interact with cpSRP54 in *C. reinhardtii* during LHCP targeting, suggesting that alternative strategies exist in algae [103]. The SRP binding principles of higher plants work similar as described for mammal and bacterial cells before. CpSRP54 consists of a N-terminal region (N domain), a central G domain which confers GTPase activity, and a methionine-rich domain in the C-terminus (M domain) [97]. The M domain of cpSRP54 lost the ability to bind SRP RNA [100], but gained a C-terminal extension containing the ARRKR motif, which determines the exclusive interaction between cpSRP54 and either cpSRP43 or the ribosome [97,104,105]. While the post-translational targeting pathway of cpSRP54 and cpSRP43 is relatively well understood, reviewed in [106], rather little is known about the co-translational function and the mechanism of target protein integration into thylakoid membranes. At least in *Pisum sativum*, binding to the ribosome is proposed to occur via the M domain interacting with the surface-exposed part of uL4c, a ribosomal protein of the large subunit (Figure 2) [107]. For many years cpSRP54 was only known to co-translationally associate with the photosystem II (PS II) core subunit PsbA and the cytochrome *b*_6_*f* complex subunit PetB [108,109,110]. An elegant recent ribosome profiling study comparing thylakoid association of translating ribosomes in *A. thaliana* wild-type and *srp54* mutant lines greatly extending the pool of putative cpSRP54 substrates [107]. This ribosome profiling approach showed that the loss of cpSRP54 does not generally result in different footprint yields or altered translational output of all plastid open reading frames (ORFs), but there was a clear decrease in membrane protein footprint yield of putative substrate proteins, showing the importance of cpSRP54 for protein integration into thylakoid membranes. Among the putative nascent polypeptides that are co-translationally targeted to thylakoid membranes are the PS I core subunits PsaA and PsaB, the PS II subunits PsbA, PsbB and PsbD, the above mentioned cytochrome *b*_6_*f* complex subunit PetB and the NADH dehydrogenase-like complex subunit NdhD [107]. This goes in hand with the previous observation that chloroplast protein synthesis of nascent integral thylakoid proteins seems to start within the soluble stroma and ribosomes are translocated to thylakoid membranes once the first transmembrane segment emerges from the ribosomal exit tunnel [111]. Once the RNC-SRP complex docks to the thylakoid membrane via binding to the cpFtsY receptor (Figure 2), the M domain of cpSRP54 is hypothesized to be repositioned towards the peptide exit tunnel to facilitate translocation of the nascent chain through the translocon. In chloroplasts the translocon consists of a reduced Sec machinery (cpSecY/E), which interacts with the integrase Alb3 for special substrates (Figure 2) reviewed in [24,106,112,113]. As there is not much structural information available about the specific interactions between cpSRP54, the ribosome, cpFtsY or the translocon, it is still unclear when exactly GTP hydrolysis occurs, how the ribosome-nascent chain complex is exactly positioned at the translocon and how nascent polypeptides are co-translationally integrated into thylakoid membranes.

In bacteria, another translocation pathway, which recruits nascent outer membrane proteins and periplasmic proteins, involves the ATPase motor protein SecA and the chaperone SecB [114,115,116]. In chloroplasts, only SecA is found. SecA seems to directly bind chloroplast ribosomes [60] but is also involved in post-translational translocation of imported proteins [24]. The only known co-translationally-bound substrate is Cytochrome *f* (encoded by petA). PetA is special since it is the only chloroplast-encoded protein with a cleavable N-terminal cpSecA-dependent signal sequence, and ribosomes translating petA seem to translocate earlier to thylakoid membranes compared to ribosomes translating putative substrates of the cpSRP54 pathway (emergence of 100 amino acids versus emergence of the first transmembrane segment) [117,118,119,120].

## 5. Ribosome Binding Sites of Co-Translationally Acting Factors

During protein synthesis, ribosomes do not act as mere static machines that translate the linear genetic code into polypeptide chains. Rather, ribosomes act as first initiator of protein folding and early protein quality control, reviewed in [3,4,9]. After synthesis at the peptidyl-transferase center (PTC), nascent polypeptides emerge through the peptide exit tunnel, which traverse across the large 50S ribosomal subunit. The tunnel has a length of 80–100 å, with an average diameter of 15 å, and it is predominantly formed by the ribosomal RNA (rRNA). Approximately 30 å apart from the PTC is the narrowest section of the tunnel, the so called “constriction zone” of 10 å in diameter. The constriction zone is formed by rRNA and two ribosomal proteins, uL4 and uL22. In contrast to previous ideas, the tunnel does not perform an inert surface but rather actively participates in recognizing the nature of polypeptides, by allowing folding of initial α-helical nascent polypeptide conformations or by inducing translational pausing, which is directly communicated to the PTC [122,123]. The tunnel exit site is constituted by rRNA and the four conserved ribosomal proteins uL22, uL23, uL24, and uL29 and serves as an important binding platform for nascent polypeptide-processing factors, such as modifying enzymes, chaperones and sorting factors. The binding sites do overlap for several of these factors, suggesting a competitive binding throughout protein synthesis. Of these ribosomal proteins, uL23 might be the most universal binding site, serving as docking site for factors such as trigger factor, SRP54 and Sec61 reviewed in [124]. In bacteria, binding of TF and L23 is mediated through the conserved ribosome binding motif within the N-terminal domain of the chaperone (Figure 1) [35]. This binding motif seems also conserved within the sequence of chloroplast TIG1s of land plants, whereas the plastidic TIG1 ribosome binding motifs of red and green algae are less conserved. Interestingly, the corresponding amino acid composition of uL23c involved in the interaction is also more diverse in algae, suggesting that the direct ribosome contact is less pronounced in algae or that the protein-protein interface has undergone co-evolution [46]. For cpSRP54, the chloroplast ribosome binding site seems surprisingly deviant compared with the situation in bacteria. Interestingly, not all plant species express the chloroplast-encoded bacterial uL23c. Instead, species of the *Caryophyllidae* and *Rosidae* families express a nuclear-encoded, eukaryotic-type uL23c, which is imported into plastids [125,126]. However, recent high-resolution structures of chloroplast ribosomes from spinach revealed that incorporation of this eukaryotic-type uL23c leads to profound changes concerning the architecture of the ribosomal tunnel exit site. Most importantly, a structural hairpin is absent, which would circumvent the binding of cpSRP54 if the interaction was of similar nature as observed in bacteria [52,127,128]. Interestingly, it was recently shown that the ribosomal interaction of cpSRP54 rather involves uL4c instead of uL23c [107]. This interaction utilizes the hydrophobic groove and the C-terminal tail region of the M domain of cpSRP54 to bind to the globular part of uL4c, which is exposed to the surface. Interestingly, there is an extended loop of uL4c, which reaches deep into the 50S subunit forming part of the constriction zone of the peptide tunnel [129,130]. Thus, uL4c might serve as nexus sensing the nature of emerging nascent polypeptides and transmitting the information to cpSRP54. This might be essential for the timed binding of cpSRP54 to chloroplast ribosomes, translating putative cpSRP54 targets, since there exists no evidence yet for SRP-mediated translational stalling in chloroplasts [107,111]. The binding position might also provide enough steric freedom that the M domain of cpSRP54 repositions towards the peptide exit tunnel to facilitate nascent chain translocation, once the SRP-ribosome-chain complex docked to the thylakoid membrane [107]. Future structural data of isolated chloroplast ribosomes in complex with associated factors are required to dissect the nature of interaction of co-translationally acting chaperones and sorting factors.

## 6. Conclusions and Outlook

Over the last years, ribosome biology, the process of translation and subsequent nascent chain maturation gained major attention in multiple disciplines of biology. This is mainly caused by the fact that ribosomes have been recognized as dynamic hubs for quickly integrating environmental stimuli and for serving as platforms for protein folding and quality control. In addition, the processes of maintaining protein homeostasis are now recognized as essential nodes for health and functionality of all organism. Still, many questions remain unsolved. For example, how is the spatiotemporal distribution of translating ribosomes organized within cells? How do ribosome-assemblies and their regulating factors differ during translation of the diverse mRNA pool? In chloroplasts of *C. reinhardtii* for example, it is now well established that protein synthesis of PS I and II subunits is orchestrated in so-called translation zones (T-zones) [131,132,133]. However, how are ribosomes organized in this sub-plastidic region, is not clear to date. In the context of translocation, the co-translational contribution of cpSRP54 and SecA remains to be shown. Furthermore, recent studies elucidated that protein complex assembly is not the result of random collision of diffusing complex subunits but assembly rather starts already during translation. Here, one dedicated subunit seems to facilitate the assembly by co-translationally interacting with unstable complex partners as soon as interaction domains emerge from ribosomes, reviewed in [3]. A similar mechanism could be envisioned in chloroplasts. Furthermore, we know little about how co-factors are co-translationally integrated and how the respective enzymes affect kinetics of protein synthesis. Given the central importance of chloroplast-encoded proteins for plant performance and hence crop yields, there is a pressing need to better understand acclimation processes in chloroplasts. This is also essential for engineering improved and more stress-resistant crop plants.

## Figures and Tables

**Table 1:** Summary of co-translationally acting factors in prokaryotic and eukaryotic cells.

	Prokaryotes	Eukaryotes		
Category		Cytosol	Chloroplasts	Mitochondria
**Sorting factors**	SRP54 (protein & 4.5S RNA) SecA/SecB	SRP54 (protein & 7S RNA) SND1 ^1^	cpSRP54 (protein only) SecA	unknown
**Ribosome-associated chaperones**	Trigger factor	Hsp70 RAC ^2^ (Hsp40 & Hsp70) NAC ^3^ (complex of α & β subunits)	Trigger factor	No trigger factor, others unknown
**Nascent polypeptide binding chaperones**	Hsp70 (DnaK) Chaperonin (GroEL/ES)	Hsp70 Prefoldin Chaperonin	HSP70B HSP90C Chaperonin	unknown

^1^ SND1 is a component of the SRP-independent targeting to the eukaryotic endoplasmic reticulum [3,4]; ^2^ RAC = ribosome-associated complex [3,4]; ^3^ NAC = nascent polypeptide-associated complex [3,4].
